# Formative Evaluation of Post-Opera Live Discussion of the Center Cannot Hold Part 2 and Resilience Workshop

**DOI:** 10.1007/s10597-024-01407-y

**Published:** 2024-12-03

**Authors:** Kenneth Wells, Juanita Booker-Vaughns, Tiffany Dzou, Elyn R. Saks

**Affiliations:** 1 Center for Health Services and Society, Jane and Terry Semel Institute for Neuroscience and Human Behavior, UCLA, Los Angeles, CA USA; 2 Department of Psychiatry and Biobehavioral Sciences, David Geffen School of Medicine, UCLA, Los Angeles, CA USA; 3 Health Policy and Management, Fielding School of Public Health, UCLA, Los Angeles, CA USA; 4 Greater Los Angeles Veterans Administration Health System, Los Angeles, CA USA; 5 Division of Cancer Research and Training, Charles R Drew University of Medicine and Science, Los Angeles, California USA; 6 The Accelerating Excellence in Translational Science (AXIS) Community Faculty, College of Medicine, Charles R Drew University of Medicine and Science, Los Angeles, California USA; 7 Department of Preventive and Social Medicine, Substance Abuse Research Training (SART) Faculty, Charles R Drew University of Medicine and Science, Los Angeles, California USA; 8 Medicine and Society Faculty for Education Students on Aging Research (ESAR) Faculty, Charles R Drew University of Medicine and Science, Los Angeles, California USA; 9 Saks Institute for Mental Health Law, Policy and Ethics, Gould School of Law, University of Southern California, Los Angeles, CA USA

**Keywords:** Arts on schizophrenia recovery, Qualitative evaluation, Audience and cast impact

## Abstract

There are few studies of impacts of arts on recovery in schizophrenia, on audience and cast responses. We developed a formative qualitative evaluation of audience and cast discussions after viewing live performances in a university setting of an opera based on Elyn Saks’ journey from psychosis, teaching law and falling in love, coupled with pre-opera workshop on community approaches to resilience. Live discussions were conducted with audience, cast members, and workshop presenters after performance of the opera, with recording and transcription, and reflexive thematic qualitative analysis sequentially conducted by 3 investigators/2 event leaders). Across 3 events, there were 81 comments (65’10” total time) from facilitator, audience members, cast and creators. Key themes across participant groups were: (1) Connecting with the story; (2) Identifying “normal” lifestyles with mental illness; (3) Refocusing goals of care for providers; (4) Increasing awareness around mental health; with an overarching theme the value of art on mental health to highlight awareness of these issues. Live art events (opera with pre-workshop) on mental health with author with lived experience present, were noted by audience and cast as enhancing connection, enhancing understanding of mental illness and clarifying goals for care, through the shared experience of art. This may inform future research and art events on mental health.

## Introduction

### Background

Schizophrenia is a complex, serious illness that is associated with social stigma affecting access to needed mental health services (Valery and Prouleau, 2022). Arts used as a resource in therapy and education may have potential to reduce stigma of mental health conditions (Estroff et al., 2004; Fancourt & Finn, 2019; Ørjasæter et al., 2017; Heenan, 2006; McLean et al., 2011; Torrissen, 2015; Lenette et al., 2016; Wilson, 2013), promoting positive attitudes toward services use (Fancourt & Finn, 2019; Hacking et al., 2006; McLean et al., 2011; Parcesepe & Cabassa, 2013; Gronholm et al., 2017), and reducing stigma of access to care (Henderson et al., 2013; Parcesepe & Cabassa, 2013). Some studies suggest potential for long-term impact (Michalak et al., 2014).

Opera is a complex art form integrating music, poetry and drama with few studies on impacts (Fancourt & Finn, 2019). In this context, the Healing and Education through the Arts (HEArts) program (Mango et al., 2018) which promotes development of new artworks on mental health, in collaboration with the UCLA National Endowment for the Arts Research Lab (https://www.NEAResearch.ucla.edu), has evaluated impact of arts events including operas through pre and post surveys and post-discussions (Skrine Jeffers et al., 2022; Bilder et al., 2022). The conceptual framework includes how artworks, including opera, may engage people by reducing stigma of mental health and increasing empathy, and through live events, increase social support, promoting positive affect and social connection (Mango et al., 2018; Bilder et al., 2022). Prior studies based primarily on pre-post surveys, suggested that operas on mental health themes can increase audience willingness to engage with persons with schizophrenia, with mediators such as empathy with largely positive qualitative comments on audience learning from an opera on schizophrenia (Skrine Jeffers et al., 2022), based on a portion of the memoire of Elyn Saks (Saks, 2007) on her journey from struggling in law school with her illness, to completing law school. Audience willingness to engage was also found in an opera on true stories of Veterans with post-traumatic stress disorder and unstable housing (Bilder et al., 2022; Wells et al., 2022, 2023). Building on this framework, we previously published a pilot evaluation based on pre and post surveys on the impact of an opera in a university medical center auditorium on positive recovery from schizophrenia: “The Center Cannot Hold Part II: Recovery” based on Elyn Saks’ memoire (Saks, 2007) from joining faculty at USC to falling in love with a law school librarian. The live events included a pre-workshop on community-partnered interventions for mental health resilience (Community Partners in Care on collaboration in care for depression, and Together for Wellness/Juntos por Nuestro Bienestar, on digital mental health resources in COVID-19, Wells et al., 2013, 2022). For the quantitative evaluation, of 107 live and 117 online attendees across 3 opera performances, 64 completed pre, 24 post, and 22 both surveys. Respondents were similar for those having pre and pre-post surveys: average age mid 50’s, 55.6% female, 10% sexual minority, 55.74% White/Caucasian, 13% Hispanic/Latino, 11% Black/African American and 20% Asian; of those pre and post surveys, 41% were providers. The pilot evaluation found statistically significant post-pre increases in positive affect (PANAS-X) and arousal (visual grid) (cites), social connectedness (Cohen’s d = 0.82 to 1.24, each *p* <.001) and willingness to socialize with someone with schizophrenia (d = 0.68, *p* =.011) (Bilder et al., 2022). The current paper provides the qualitative analysis of the post-event discussions for the 3 live performances, to supplement the survey results with the narrative experiences of audience members, cast members, presenters in the pre-opera workshop, and creators of the opera libretto and composition. The goal was to supplement the quantitative findings to understand more fully the experience of individuals involved with the live performances, the potential impacts on attitudes toward mental health and social connection, as well as clarify any concerns about the event format or content, to inform future research on impacts of art events on mental health. Thus, our purpose was to explore live audiences’ and cast members’ perceived impact from attending or performing an opera on positive recovery from schizophrenia.

### Hypotheses

We hypothesized that the workshop and opera would increase audience members’ positive affect and sense of social connection, including interacting on common issues related to the opera in post discussion. We also thought that as in prior arts events, cast members and audience members might share similar experiences, relating specifically to words/themes presented in the opera.

## Methods

### Design

This qualitative formative evaluation featured analysis of post-opera discussions by audience members, cast, facilitator and creators of the opera, as well as participants from the pre-opera workshop continuing as opera audience members. An information sheet was available for online attendees and read at all events by an investigator. Post-event discussion participation was voluntary and anonymous for persons 18 years or older (exclusion under 18), with data collection approved by UCLA’s Institutional Review Board. The workshop and opera were advertised by UCLA, cast, and community partners and Los Angeles County Department of Mental Health (LACDMH) Take Action LA for Mental Health initiative. Events were free at a neuroscience center auditorium.

### Workshop Format

The 45-minute pre-opera workshop on resiliency and recovery from mental illness for diverse populations had a community facilitator, for 2 events a director from Los Angeles County Department of Mental Health, author Elyn Saks on her journey to recovery, remarks by academic and community leads on community-partnered projects (Wells et al., 2013, 2022). The workshop concluded with a choral work “I am a Thriver!” by the opera composer on a poem by community leader Loretta Jones, available on National Academy of Medicine Visualize Health Equity website (https://nam.edu/visualizehealthequity/#/artwork/94).

### The Center Cannot Hold Part II

Recovery is a 75-minute, one-act opera, composed by a psychiatrist based on Elyn Saks’ memoire (Saks, 2007). The story features her appointment as law professor at USC, experiences of schizophrenia and treatment experiences including conflicts with her provider, different parts of herself represented by 3 characters; her journey to recovery as a law school professor and falling in love with a law school librarian.

Post-Event Discussion Groups: Each live event had a post-discussion facilitated by a community partner/faculty member. Discussions followed a guide managed by the facilitator and shared with audience on a slide.

### Post-Event Discussion Guide

- Initial impressions of the opera and workshop and what they convey?
- How has it affected you?
- Does it seem relevant to any of your experiences or impressions?
- What do you think the opera says about experience of persons with mental health challenges, their friends or colleagues?
- What does the event say about the experience of providers?
- What are your thoughts about how arts can affect our views of experiencing serious mental illness?
- Cast, speakers, creators: what has the experience meant to you?

### Qualitative Analysis

Discussions were recorded, transcribed, and de-identified for analysis (listing participant type: audience, cast, creator, facilitator; gender; performance number (P1-3) and transcription lines for quotes). Data were analyzed with reflexive thematic analysis, utilizing a six-phase approach outlined by Braun and Clarke (2022). First, two investigators (discussion facilitator; and qualitative investigator) reviewed transcripts, taking note of potential codes. Next the analytic team generated initial codes using process coding to identify participants’ reflections in the data. Subsequently, investigators compared and distilled 11 groups of overarching codes into 4 potential themes and one overarching theme. In the fourth and fifth phase of analysis, the investigators reviewed, defined, and named themes and subthemes. Finally, the research team selected *quotes* to represent themes and produced an analytic narrative reporting participants’ perceived impact of attending workshop and opera about positive recovery from schizophrenia.

## Results

For 3 live performances, there were 81 comments with total time of 65’10”, with distribution across events and participants as follows: (Performance number and discussion time, participant type and number of comments): Performance 1 (8’42”, 15 comments): Facilitator (5); Male audience member (2); Audience multiple speaking at one (3); Female audience (1); Female cast (1); instrumentalist (1); Creators (2); Performance 2 (32’03”, 37 comments): Facilitator (9); Creators (5); Post discussion speaker female (4); Female audience member (7) Male audience member (3); Creators (3); Male cast (2); Female cast (4); Performance 3 (24’25”, 29 comments): Facilitator (10); Female audience member (7); Female provider audience member (3); Male audience member (3): Creators (3); Female cast (3). For the Table 1 as well as examples below, we use Audience (A)/Provider (P), Female (F), Male (M); Cast (Ca); Workshop Speaker (WS); Facilitator (Fac); Creators (Cr).

**Table 1:** Opera Live discussion of the Center cannot hold part 2 and Resilience Workshops: Quote sources: 3 performances (P1, 2, 3), comment source: Facilitator (Fac), audience (A)/Provider(P)/Male(M)/female(F), cast(ca), Workshop Speaker(WS), creators (cr)

Key Theme(s)	Comments 6/17/2023 (P1)	Comments 6/23/2023 (P2)	Comments 6/25/2023 (P3)
[1] Connecting with the story [2] Identifying “normal” lifestyles with mental illness [3] Refocusing goals of care for providers [4] Increasing awareness around mental health [5] Overall: Impact of art/music/stories on mental health	[1, 5] “My own struggles in recent years having experienced PTSD because of a car accident; severe depression and anxiety I completely understand part of it, so I used that part of it to tap into what I can imagine Elyn has been experiencing which she has articulated so beautifully in her book and in the score; when it’s set to your music it moves people” (P1, Ca) [2] “in high school it was like 13–15 years I didn’t date because I was too tormented by internal humans; and then I started going out with (him) and everything changed” (P1, Cr). [1] “You can’t judge the person by what you see; really sit and talk with the person it’s like ‘don’t judge a book by it’s cover’” (P1, A/M) [1, 5] “Well obviously it makes a great deal to me every one time I’m in one of these operas and to have this beautiful woman sitting her in the audience it’s an honor to portray her story to the best of my ability” (P1, Ca) [1, 5] “I think for me actually seeing it play out and reading it and engaging in the music, it brought such feelings about Elyn’s experiences, her life, her love you felt it all through all the different areas of performance art here…it was amazing, to see it expressed, the visual, the auditory, all proof that connecting to my sensories and helping me to engage and be overjoyed; Elyn you are a hero to me, watching your life unfold was a great story, a great love story” (P1, Fac)	[4, 5] “I could see this evolution of opportunity that once you get the proper care around you that there is this ability to stabilize your life and desire more for something beyond just medication; just being a symptom of condition but actually living life and being better than that condition and that’s where it was just even listening to Dr. Saks, it just made my eyes wide open to see wow, this is why we do what we do, so that was amazing.” (P2, WS). [3, 4, 5] “ I’m not so knowledgeable about these different things through art, and I kind of felt heavy and sleepy during part of it, I just woke up and I found myself smiling, so I don’t know if it was just the singing and the voices, but you know to tell the story this way, I just I will walk out uplifted by your story because of the opera, it’s great. (P2. A/F). [1, 4, 5]“ It’s an effective piece, congratulations; I love that it’s like opening up a discussion about mental health, it’s really important of course. I feel like actually our stories Dr. Saks are very similar. (P2, A/F) [3, 5] “what Dr. Saks illustrates that, being in love and having a relationship, having a career, those are all the things that people want to do, and I think our focus should be less on symptom control and more on enriching someone’s life. “ P2 (WS)	[4, 5] “It’s very, very uplifting, and I’d say educational that people with serious mental illness have life and work and it was very inspirational; so music was lovely, thank you.”(P3, A/F) [3, 4, 5] “how beautiful it was to see a message of hope and optimism and fulfillment it’s so rare that we hear and see these messages in psychiatry in particular, speaking as a psychiatrist; we just need so much more of that.” (P3, A/P/F) [2, 3, 4, 5] “ I love this piece and I love your work and everything you’re doing around this, the fantastic performances; I think for opera, opera is like painting, it’s a tool it is a vehicle for us to talk about deeper issues; and it doesn’t always have to be stories about castles and lords and ladies, it can have real relevance, it can have us talking about mental health and growing and also finding love within.” (P3.A/M) [2, 4, 5] “I kept seeing the light going on and then I thought about it and there was still light, and hope because I know that when we are looking at our loved ones and others that we may know that have been diagnosed with schizophrenia and wonder we don’t understand it all, but seeing this, images, and looking at your life, seeing that there is hope and light in this life. (P3, Fac). [4, 5] “ how many could be if we devoted proper resources, and that’s exactly right; opening people’s eyes to what the illness is like and what we really need to do. (P3, Cr). [1, 3, 4, 5] “it was heartwarming, moving, I worked in the field, dealt with families with many MH issues, and have family members, one family member had schizophrenia, OK, and I have seen in many people, how the resources were not brought to bear for fulfillment, there are so much that could be for so many people, and I thought that this was a very telling presentation.” (P3, A/F) [1, 2, 5] “When we encounter people and the stereotypes dissolve; but we don’t always encounter everyone in our lives and through art it’s like one step removed but it gives us access, to other’s experience and hopefully it will help bring break down prejudice and fear” (P3, A/F)

### Results from Thematic Analysis

Four main themes were identified from this thematic analysis: (1) Connecting with the story; (2) Identifying “normal” lifestyles with mental illness; (3) Refocusing goals of care for providers; (4) Increasing awareness around mental health, with example statements in the Table and subthemes discussed below. An overarching theme 5 was value of art/music/stories on mental health to highlight awareness of all themes.

Theme 1: Connecting with the story. Participants reflected deep, personal connections with the story from the opera. This was represented by three properties/subthemes: (1) Relating to the story; (2) Increasing family member’s insights into mental illness; (3) Stepping into the shoes of someone with mental illness.

Relating to the story: Participants found the story to be relatable to their own life experiences, such as similar struggles with mental health, including anxiety, depression, and suicidality, and how the story was relatable to their experiences. One example of relating to the opera: My own struggles in recent years having experienced PTSD because of a car accident… so I used that part of it to tap into what I can imagine Elyn has been experiencing which she has articulated so beautifully in her book and in the score.”(P1, Ca).

Increasing family member’s insights: Participants shared experiences such as being a provider for families with mental health issues or having family members with mental illness with the opera increasing awareness of understanding: “I dealt with families with many mental health issues, and have family members, on had schizophrenia, there is so much that could be for so many people and I thought this was very telling, when things come together.” (P3, A/F).

Stepping into the shoes of someone with mental illness: Participants referred to how the story of the opera: “.

gives people a window into the mind of someone suffering from psychosis may help them understand more and be more empathetic.” (P2, Cr).

Theme 2: Identifying “normal” lifestyles with mental illness. Participants noted that the story highlighted recovery and having normal lifestyles. Key subthemes: 1), growing from a history of mental illness and limitations into normal activities; and 2) realizing normal life is possible from hearing true stories.

Growing from a history of mental illness and limitations into normal activities: One participant shared their experience as an adolescent in high school “I didn’t date because I was too tormented by internal humans” and then started going out “and everything changed.” (P1, Cr). Another said: “it can have us talking about mental health and growing and also finding love within very, very tragic details of our lives sometimes.” (P3, A/M).

Realizing that normal life is possible from hearing true stories: One participant highlighted having events for sharing of stories: “it was the story that was affecting and that you could relate to, in dealing with modern music the stories that the operas that I really like are those that I can relate to.” (P1, A/M) Another noted how shared lived experience informed personal experience: “it awoke something in me that I had forgotten about because I also feel that my life is thriving as well.” (P2, A/F).

Theme 3: Refocusing goals of care for providers: Both providers and community members commented on how the opera/workshop reinforced important changes in providing services for mental illness, with key examples/subthemes: (1) importance of hope; (2) enriching someone’s life; 3)importance of broadening resources.

Importance of hope: One provider noted: how beautiful it was to see a message of hope and optimism and fulfillment it’s so rare that we hear and see these messages in psychiatry. (P3, A/P/F)

Enriching someone’s life: Reflecting on messages from the opera, one participated noted: “our focus should be less on symptom control and more on enriching someone’s life. “( P2, S).

Importance of broadening resources: One participant noted that, as in the opera story, they had seen many for whom resources “were not brought to bear for fulfillment, there are so much that could be for so many people, and I thought that this was a very telling presentation.” (P3, AF) Another noted “that there is this ability to stabilize your life and desire more for something beyond just medication…actually living life and being better than that condition.”(P2, S) Another said: “I don’t think anybody comes into treatment with a psychiatrist wanting to just reduce the voices, wanting to reduce suicidality, they want to live a life.” (P2, S).

Theme 4: Increasing awareness around mental health: Participants highlighted issues of: (1) importance of understanding mental illness; (2) the path forward; and (3) understanding people suffering from schizophrenia.

Importance of understanding mental illness: One participant noted: “it’s like opening up a discussion about mental health, it’s really important of course.” (P2, A/F).

The path Forward: A participant indicated that the work was “opening people’s eyes to what the illness is like and what we really need to do.”(P3, Cr) Another said it was “educational that people with serious mental illness have life and work; and it was very inspirational.” (P3, A/F).

Understanding People Suffering from Schizophrenia: One person said that opera was “a window into the mind of someone suffering from schizophrenia, gives hope to those who suffer from schizophrenia and understanding to those who don’t.” (P2, Cr).

Overaching Theme 5: Value of art/music/stories on mental health to highlight awareness: Most participants referred directly to the event. One noted: “come into the theater and that’s what’s so magical, it’s live and you can feel it and so when you get the lived experience.” (P3, A/M) Another noted: “Telling the story gives people a window into the mind of someone suffering from psychosis may help them… be more empathetic.” (P2, Cr) Another noted: “looking at music or theater or opera, to demystify threat or normalize something, through the eyes or experience of someone else, you get to see something in a real way.” (P2, S). Another: “through art it’s like one step removed but gives us access, to other’s experience and hopefully it will help bring break down prejudice and fear.” (P3, A/F) One noted “I love the sound of music, so it helps me, soothes me.” (P2,S) The breadth of impact is noted in this comment: “when you put a story like this to music it takes away gatekeeping that we normally have around these issues, if we can all allow music to wash over us with this difficult and troubling subject matter at times, but also inspirational and hopeful, it does help to remove the stigma.” (P3, Ca).

## Discussion

This pilot study used reflexive thematic qualitative analysis to understand the impact on audience members and cast of an arts event (opera) on recovery from schizophrenia, based on lived experiences of a law professor who started teaching and coping with her illness and progressing to falling in love. The analysis used transcripts of recordings of post-discussions after live performances. Each of three opera performances was preceded by a workshop on community resilience. The events were held in a university medical center auditorium, with an audience that included many medically trained members and some with lived experience, which could suggest that audience members were familiar with mental health issues.

The qualitative analysis revealed four key themes and one overarching theme, many of which were shared by community members and providers. The themes included connecting with the opera’s story on lived experiences, including identifying “normal” lifestyles with mental illness, a variant of sharing stories; refocusing goals of care for providers, such as understanding some difficulties in getting needed care and broadening care to support lived experience, and also the opera’s impact on increasing knowledge/understanding and importance of mental health/illness in society. These themes were largely shared in a personal way almost like a “group therapy” session, given the connection people seemed to feel and articulate after the arts event/workshop. Serious mental illness is an important health condition (Kessler et al., 2005; Wu et al., 2006). Across themes, an overall theme was that presenting true stories of recovery through art (with the individual/author present) may increase positive affect and sense of belonging as well as public understanding.

These findings are consistent with the hypotheses that the event would increase positive affect, social connection, including sharing on common issues related to the opera/workshop, across audience and case members. This can be an important preliminary finding for the value of art on serious mental illness, given high social stigma of schizophrenia (Valery and Prouleau, 2022), with evidence on art therapy promoting positive attitudes toward resources (Fancourt & Finn, 2019), but now expanded to public arts on lived experience. Important next research steps could be exploring longer-term effects on stigma and accessing services for such public events, given studies suggestion potential for longer-term impact (Michalak et al., 2014).

### Limitations

Given location in a healthcare setting with providers present as well as community members and author with lived experience in post-discussion, this context may have enhanced shared knowledge, as participatory artworks have been shown to impact stigma (Yotis et al., 2017; ) This context could have stimulated positive responses and audience sharing experiences, and it would be important to present and evaluate similar activities in broader public settings. Sample sizes (*N* = 81 discussions) were moderate but fairly large for qualitative analysis. There were no comparison conditions, so attributions to characteristics of story or production are exploratory, largely as attributed directly by discussion participants.

## Conclusion

Overall, the post-discussions of the live performance suggested that audience and cast members connected to the story, found it relevant to their experience and stimulated sharing of experiences, informing the community members and having impact on providers and their framing of care goals. A common issue across themes was the impact of arts/music/stories for engagement in understanding mental illness with empathy and connection, which may inform development and evaluations of arts-initiatives on serious mental illness.
